# Complexity Analysis of Global Temperature Time Series

**DOI:** 10.3390/e20060437

**Published:** 2018-06-05

**Authors:** António M. Lopes, J. A. Tenreiro Machado

**Affiliations:** 1 UISPA–LAETA/INEGI, Faculty of Engineering, University of Porto, Rua Dr. Roberto Frias, 4200–465 Porto, Portugal; 2 Department of Electrical Engineering, Institute of Engineering, Polytechnic of Porto, R. Dr. António Bernardino de Almeida, 431, 4249–015 Porto, Portugal

**Keywords:** complexity, Lempel–Ziv complexity, sample entropy, temperature time series

## Abstract

Climate has complex dynamics due to the plethora of phenomena underlying its evolution. These characteristics pose challenges to conducting solid quantitative analysis and reaching assertive conclusions. In this paper, the global temperature time series (TTS) is viewed as a manifestation of the climate evolution, and its complexity is calculated by means of four different indices, namely the Lempel–Ziv complexity, sample entropy, signal harmonics power ratio, and fractal dimension. In the first phase, the monthly mean TTS is pre-processed by means of empirical mode decomposition, and the TTS trend is calculated. In the second phase, the complexity of the detrended signals is estimated. The four indices capture distinct features of the TTS dynamics in a 4-dim space. Hierarchical clustering is adopted for dimensional reduction and visualization in the 2-dim space. The results show that TTS complexity exhibits space-time variability, suggesting the presence of distinct climate forcing processes in both dimensions. Numerical examples with real-world data demonstrate the effectiveness of the approach.

## 1. Introduction

Understanding climatic variability is extremely important nowadays. The Earth’s climate is changing and we are facing an increasing number of extreme weather events, like floods, droughts and anomalous temperatures. Since the beginning of the 20th century the Earth’s surface average temperature has increased by nearly 0.8 °C [1,2,3], and several projections based on climate models estimate that during this century, the average temperature may increase by between 1.1 °C and 6.4 °C [1]. It has been verified that the global surface temperature has risen at an average rate of about 0.17 °C per decade since 1970 [1]. Moreover, the annual mean Arctic sea-ice extent decreased at a rate of about 3.5% to 4.1% per decade over the period from 1979 to 2012 [4]. The Earth is warming and it seems that human activity is one of the main causes for this phenomenon [5,6]. Estimates from the Intergovernmental Panel on Climate Change (IPCC) revealed a considerable increase of anthropogenic greenhouse gas emissions since the pre-industrial era [1]. In the period from 1970–2010, the annual growth rate of emissions was above 1.3% [7]. Some impacts of global warming, such as record high temperatures, melting glaciers and severe flooding are becoming increasingly common around the world [8,9,10,11,12]. Besides the direct effect on temperature, warming leads to the modification of wind patterns, the development of humidity, and significant changes in the rates of precipitation. These phenomena are under intensive research due to their major impacts on the social, economic and health aspects of human life [13,14,15,16].

The analysis of temperature time series (TTS) is an important topic that can help us understanding better climate dynamics. Several indicators of climate change can be used, but the time evolution of the Earth’s surface temperature is probably the more straightforward measure to follow [17]. Furthermore, reliable temperature data records comprising information from meteorological stations around the world are available to support data processing [18,19].

We can find in the literature several strategies for analyzing the TTS [20]. Grieser et al. [21] studied the monthly mean temperatures of European meteorological stations. They applied statistical tools to decompose the TTS into significant components and found that the phase of the annual cycle shifts within the year, backwards and forwards in Western and Eastern Europe, respectively. The authors observed that the occurrence of extreme events increased significantly and corresponded mostly to cold peaks in winter. They also found large harmonic components with a period of 92.3 months in data from Northern and Western Europe. Additionally, this period, corresponding to about 7.7 years, was also observed in the North Atlantic Oscillation. Hughes et al. [22] pointed out that the Antarctic Peninsula region is warming faster than the rest of the world. They analyzed the minimum and maximum TTS at the Faraday/Vernadsky (Latitude 65.25 S, Longitude 64.25 W) station using a multiple regression model with non-Gaussian correlated errors. It was calculated that there was an increase in the minimum monthly temperature of 6.78 °C during the years 1951–2003. Viola et al. [23] adopted nonlinear processing tools to analyze TTS. The method of delay coordinates was applied for state space reconstruction and the delay parameters were determined by means of two methods, namely the average mutual information and the false nearest neighbors. Temperatures were extrapolated until 2028 by means of simple nonlinear prediction, which, after the state space reconstruction, uses a limited number of neighboring samples to perform predictions. Founda et al. [24] employed linear regression fitting to analyze the air TTS in Athens from 1897 until 2001 and observed a tendency towards warmer years. Ge et al. [25] studied the temperature variation in China over 20 centuries, revealing not only the cold periods from 1620–1710 and 1800–1860, but also the warming registered during the 20th century. Deser et al. [26] examined the trends of the sea surface and marine air temperatures and observed warming in all cases, with the exception of the Northwestern Atlantic Ocean. The largest warming trends were found in the middle latitudes of both the Northern and Southern hemispheres. Oñate and Pou [27] processed TTS from eleven meteorological stations on the Iberian Peninsula. Trends were identified by means of the Mann–Kendall test, and multidimensional scaling provided automatic clustering. More recently, Stephenson and Doblas-Reyes [28] used multidimensional scaling as an exploratory tool to describe ensembles of forecasts. Multidimensional scaling is a computational technique for exploring similarities between groups of data and easing the visualization of hidden patterns [29]. Lopes and Machado [5] adopted multidimensional scaling to study complex correlations between global TTS.

Earth surface temperatures exhibit slow space-time system dynamics that embed long memory effects and complex relationships. Climate data can be analyzed in a straightforward manner by calculating the means and variances for different levels of spacial and temporal aggregation, but these methods neglect long range dynamic phenomena. Furthermore, while many studies have addressed the problem of global warming, the aspect of the increasing complexity of the data has been somewhat overlooked. In fact, the complexity of the TTS is embedded into the measurements, but needs to be tackled independently of the problem of increasing temperatures. The complexity point of view may lead to a deeper insight into the predictability of future TTS evolution.

In this paper, global TTS are viewed as manifestations of a dynamic system. For each TTS, the trend is calculated by means of empirical mode decomposition (EMD), and the complexity of the detrended series is assessed by means of four indices, namely the Lempel–Ziv complexity (LZC), the sample entropy (SampEn), the signal harmonics power ratio (η), and the fractal dimension (FracDim). The four indices capture distinct features of the TTS in a multidimensional space that is projected in 2D through hierarchical clustering. The space-time variability of the complexity measures suggests that the inputs of climate vary in both dimensions. In particular, the increasing complexity of the TTS in recent years shows that temperatures are becoming more difficult to predict. Numerical examples using real-world data for one century as well as the four complexity indices, illustrate the effectiveness of this new point of view for tackling TTS.

With these ideas in mind, the paper is organized as follows. Section 2 presents the main fundamental issues that influence global warming. Section 3 overviews the mathematical tools used for processing data. Section 4 and Section 5 analyze the global TTS complexity by means of different indices. Finally, Section 6 outlines the conclusions.

## 2. Mathematical Fundamentals

This section introduces the main mathematical tools adopted for data processing, namely the LZC, SampEn, Fourier series (FS), EMD, and FracDim. These tools are well suited to the time series generated by many natural phenomena, such as climatic, geophysical and biological processes.

### 2.1. Lempel–Ziv Complexity

The LZC is a method of symbolic sequence analysis that measures the complexity of finite time series [30,31,32]. The LZC is based on computing the number of distinct subsequences and their recurrence rates along the series of data [33,34,35,36].

Let us consider the finite time series, x(k), where k=1,2,…,K represents the discrete sampling instants. In the first phase, x(k) is converted into a symbolic binary sequence, S={s(1),s(2),…,s(K)}, such that

(1) $$s\left(k\right)=\left\{\begin{array}{c} 0,\;if\;x\left(k\right)<T_{h}\\ 1,\;if\;x\left(k\right)\geq T_{h} \end{array}\right.,$$

where, $T_{h}$ denotes a pre-defined threshold value. Usually the median of x(k) is adopted for $T_{h}$, since it performs robustly against outliers [37]. In a second phase, we parse the original sequence, *S*, from left to right, to obtain a new (parsed) sequence, $S^{\prime }$, formed by distinct subsequences or words. To obtain $S^{\prime }$, different parsing methods are available [38,39,40]. Herein we summarize the original scheme proposed by Lempel and Ziv [30,41].

Let S(p,q) denote a subsequence of *S* composed of the symbols between positions *p* and *q* (in our case, time instants). Thus, we have S(p,q)={s(p),s(p+1),…,s(q)}, for q≥p, and S(p,q)={} (i.e., the empty set), for q<p. The parsed sequence, $S^{\prime }$, is obtained as follows:

1. Initialize:
  (a) The parsed sequence, $S^{\prime }=\left\{s\left(1\right)\right\}$;
  (b) An auxiliary sequence, Q={};
  (c) A pointer, θ, such that, θ(1) points to the symbol, s(1), of *S*;
  (d) p=1.
2. At the iteration *p*:
  (a) Advance from θ(p) to θ(p+1) and extend the auxiliary sequence, *Q*, by appending the symbol s(p+1);
  (b) Check whether the current auxiliary sequence *Q* matches any subsequence of S(1,p). If no match is found, then append, as a new word, the auxiliary sequence, *Q*, to the parsed sequence, $S^{\prime }$, and reset to Q={};
3. Increment *p*;
  (a) If p<K, then go to step 2;
  (b) If p=K, then append *Q* to $S^{\prime }$ to yield the final parsed sequence;
4. Count the number of different words, c(K), in $S^{\prime }$.

To obtain a complexity measure independent of the length of *S*, the count value c(K) is normalized, yielding [30]

(2) $$LZC=\frac{c\left(K\right)\log_{2}\left(K\right)}{K},$$

which reflects the rate of occurrence of new patterns in *S*.

### 2.2. Sample Entropy

The SampEn is a modified version of the “approximate entropy” [42], used to measure complexity. It is defined as the symmetrical version of the logarithm of the conditional probability of two sequences that are similar for *m* points, remain identical for m+1 points. The value of SampEn has little variation across the length of a time series, yielding a characteristic measure of its complexity [43]. The SampEn can be calculated by the five steps presented below [44,45,46,47,48]:

1. Generate a set of *m*-dimensional vectors, $X_{p}^{m}$,
  (3) $$X_{p}^{m}=\left[x\left(p\right),x\left(p+1\right),\ldots ,x\left(p+m-1\right)\right],\;1\leq p\leq K-m+1,$$
  representing *m* consecutive values of x(k), starting at point *p*;
2. Determine the similarity, $D_{p,q}\left(r\right)$, between $X_{p}^{m}$ and $X_{q}^{m}$ (i.e., the vector representing *m* consecutive values of x(k), starting at point *q*), by calculating
  (4) $$D_{p,q}\left(r\right)=\left\{\begin{array}{c} 1,\;if\;d_{p,q}<r\\ 0,\;if\;d_{p,q}\geq r \end{array}\right.,$$
  where $d_{p,q}=\max_{n=0,1,\ldots ,m-1}\left|x\left(p+n\right)-x\left(q+n\right)\right|$ is a distance, and *r* is a tolerance value;
3. Compute the correlation sum, $B_{p}^{m}\left(r\right)$, under the constraint p≠q, to exclude self-matches,
  (5) $$B_{p}^{m}\left(r\right)=\frac{\sum_{q=1,q\neq p}^{K-m}D_{p,q}\left(r\right)}{K-m-1};$$
4. Find the probability, $B^{m}\left(r\right)$, of template matching for all vectors by
  (6) $$B^{m}\left(r\right)=\frac{\sum_{p=1}^{K-m}B_{p}^{m}\left(r\right)}{K-m};$$
5. Calculate the SampEn as
  (7) $$SampEn=-\ln\frac{B^{m+1}\left(r\right)}{B^{m}\left(r\right)}.$$

The values of the parameters *m* and *r* are important in determining the SampEn, but no exact guidelines exist for optimizing their values. We know that the accuracy and confidence of the SampEn estimates increase when a small *m* (short template) and large *r* (wide tolerance) [42,46] are chosen. Moreover, when *r* is too small, poor conditional probability estimates are obtained, while when *r* is too large, detailed information is lost and SampEn tends to be 0. To avoid problems caused by noise *r* must be chosen carefully [42,46]. The generally-adopted parameter values are m=1 or m=2, and r=ρσ, where σ denotes the standard deviation of the original time series, x(k), and ρ∈[0.1,0.25] [46].

### 2.3. Fourier Analysis

The classical FS was proposed by Joseph Fourier (1768–1830) when studying problems in the area of heat conduction. The FS expresses a given signal by means of an orthonormal basis of trigonometric functions.

Let us assume that x(k)=x(k+T), k,T∈N, is a periodic function with period *T*, $T=\frac{1}{f}$, and ω=2πf, where *f* and ω denote the frequency and angular frequency, respectively. The signal x(k) can be expressed by means of FS expansion [49,50]:

(8) $$x\left(k\right)=\sum_{n=0}^{T-1}a_{n}e^{j2\pi fnk},$$

where $j=\sqrt{-1}$, and the Fourier coefficients, $a_{n}$, are given by

(9) $$a_{n}=\frac{1}{T}\sum_{k=0}^{T-1}x\left(k\right)e^{-j2\pi fnk}.$$

According to Parseval’s theorem, the average power of x(k) is given by

(10) $$P_{x}=\frac{1}{T}\sum_{k=0}^{T-1}{\left|x\left(k\right)\right|}^{2}=\sum_{n=0}^{T-1}{\left|a_{n}\right|}^{2}.$$

### 2.4. Empirical Mode Decomposition

The EMD is a powerful signal processing tool that is able to deal with non-stationary and non-linear time series [51,52,53]. The basic idea is that the original signal x(k) is decomposed into a finite set of simpler signals called intrinsic mode functions (IMF). The algorithm is as follows [51]:

1. Identify all extrema of x(k);
2. Interpolate between minima and maxima, and find the lower and upper envelopes, $e_{min}\left(k\right)$ and $e_{max}\left(k\right)$, respectively;
3. Calculate the mean using $m\left(k\right)=\frac{1}{2}\left[e_{min}\left(k\right)+e_{max}\left(k\right)\right]$;
4. Extract the details using d(k)=x(k)−m(k);
5. Iterate on the residual, m(k).

For real-world data, this procedure iterates steps 1–4 until the detail signal, d(k), is an intrinsic mode function (IMF), meaning that it has a zero-mean according to some criterion. It should be noted that the complete process guarantees that the number of extrema decreases between two successive residuals and that the decomposition is achieved with a finite number of modes.

The original signal can be represented by

(11) $$x\left(k\right)=m_{L}\left(k\right)+\sum_{l=1}^{L}d_{l}\left(k\right),$$

where $d_{l}\left(k\right)$ represent the modes and $m_{L}\left(k\right)$ denotes the residual.

### 2.5. Fractal Dimension

A fractal is a geometrical object that displays identical patterns at different scales. The FracDim is a measure of how much the fractal fills the space as it is magnified from larger to smaller scales [54,55,56]. The box-counting method is often adopted for estimating the FracDim, due to its straightforward numerical implementation. For a fractal represented by a binary image, with non-zero pixels belonging to the fractal, and zero pixels forming the image background, the box counting algorithm is as follows [56]:

1. Pad the image with background pixels so that its dimensions are at a power of 2;
2. Cover the fractal object with a grid of squares with size ϵ (in the first iteration, there is just one square of equal size to the size of the image);
3. Count the number of boxes (i.e., squares), $N_{\epsilon }\in N$, needed to cover the object;
4. If ϵ>1, then make $\epsilon \leftarrow \frac{\epsilon }{2}$ and repeat step 2.
5. Estimate the FracDim as the slope of the log-log plot, $N_{\epsilon }$ versus ϵ, calculated by means of the least squares method.

The estimate of FracDim obtained by the algorithm is a good approximation to

(12) $$FracDim=-\lim_{\epsilon \to 0^{+}}\frac{\log N_{\epsilon }}{\log\epsilon }.$$

## 3. Dataset

The University of Delaware “Terrestrial Air Temperature: 1900–2014 Gridded Monthly Time Series (V 4.01)” was used. The dataset is publicly available at the website http://climate.geog.udel.edu/~climate/html_pages/download.html#T2014, and was retrieved on 1 March 2018. The archive consists of monthly mean air TTS for the period from January 1900 up to December 2014. The spatial coverage corresponds to land areas, with one TTS per cell, on a $0.5^{\circ }\times 0.5^{\circ }$ latitude × longitude grid of resolution. The dataset was compiled by means of interpolation techniques on data gathered from several sources, including the Global Historical Climatology Network Monthly Version 3 (GHCN3) dataset [18]; the Daily Global Historical Climatology Network (GHCN-Daily) archive [57]; the Atmospheric Environment Service/Environment Canada archive; records from the State Hydrometeorological Institute of St. Petersburg, Russia; data for Greenland provided by the GC-Net [58]; records from the Automatic Weather Station Project (Charles R. Stearns, University of Wisconsin—Madison); the Global Synoptic Climatology Network archive (Dataset 9290c, of the National Climatic Data Center); and observations contained within the Global Surface Summary of Day (GSOD) [59].

Herein, we illustrate the usability of the dataset, while highlighting some aspects of the global warming revealed by the temperature anomalies [13]. Given a reference period, $P_{12}=\left[P_{1},P_{2}\right]$, from year $P_{1}$ up to year $P_{2}$, the temperature anomaly for the period $P_{34}=\left[P_{3},P_{4}\right]$ is

(13) $$A_{12}^{34}=\frac{1}{P_{4}-P_{3}}\sum_{n=P_{3}}^{P_{4}}y\left(n\right)-\frac{1}{P_{2}-P_{1}}\sum_{n=P_{1}}^{P_{2}}y\left(n\right),$$

where y(n) represents the average temperature of year *n*.

**Figure 1:**
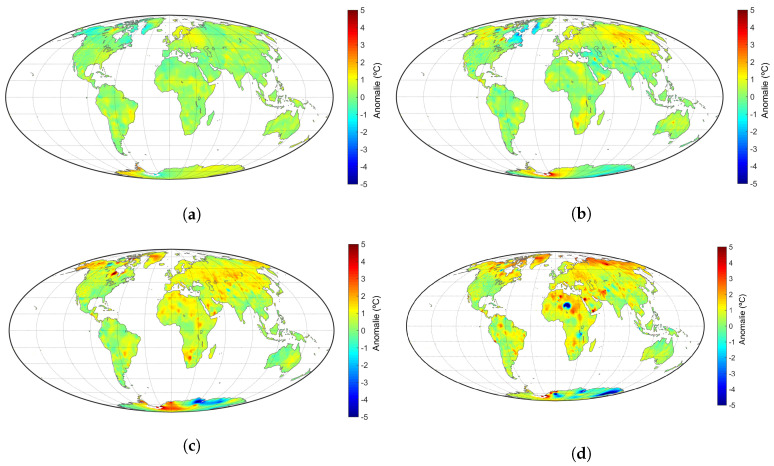
Worldwide land temperature anomalies relative to $P_{12}=\left[1951,1980\right]$ for the 5-year periods: (**a**) $P_{34}=\left[1970,1974\right]$; (**b**) $P_{34}=\left[1990,1994\right]$; (**c**) $P_{34}=\left[2000,2004\right]$; (**d**) $P_{34}=\left[2010,2014\right]$.

Temperature anomalies represent deviations from a reference value, or long-term average [13]. Positive (negative) anomalies mean that the observed temperatures are higher (lower) than the reference value. Figure 1 depicts the annual land temperature anomalies for the years $P_{34}=\left[1970,1974\right]$, $P_{34}=\left[1990,1994\right]$, $P_{34}=\left[2000,2004\right]$ and $P_{34}=\left[2010,2014\right]$, relative to the period $P_{12}=\left[1951,1980\right]$. The results show the Earth’s warming clearly, with emphasis on certain regions, namely in the Northern Hemisphere.

## 4. On the Complexity of TTS

The TTS, x(k), k=1,…,K, exhibits a trend. Here, *k* represents time with a 1-month resolution, and k=1 and k=K=1380 correspond to the initial and final instants—January 1900 and December 2014, respectively. The EMD proved to be a good method for determining the trends of data from nonstationary and nonlinear processes, without the need to know the trends’ functional forms [53]. Within this approach, the TTS trend was estimated by the residuals of the EMD.

**Figure 2:**
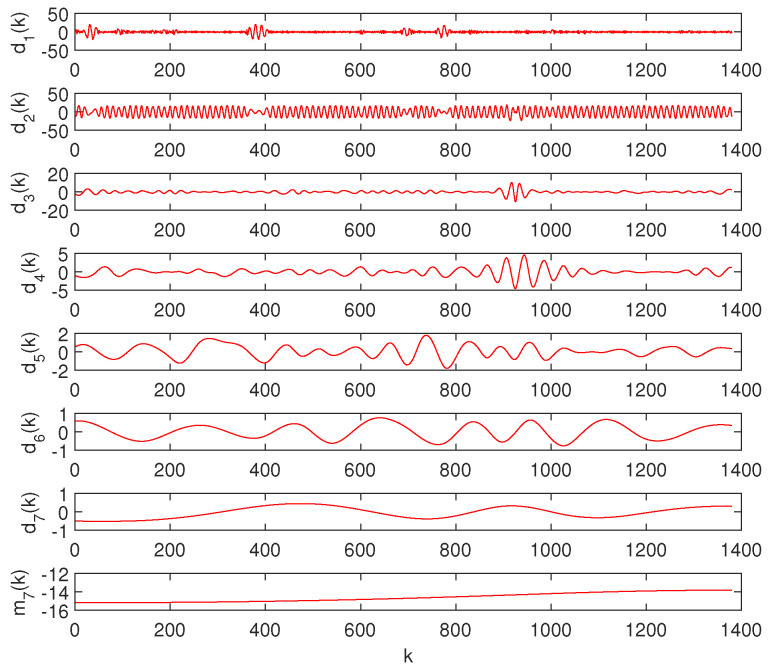
The intrinsic mode functions (IMF), $d_{l}\left(k\right)$, l=1,…,7, and the residuals, $m_{7}\left(k\right)$, obtained for a temperature time series (TTS) of a station with latitude/longitude coordinates $\left(71.25^{\circ },89.75^{\circ }\right)$.

**Figure 3:**
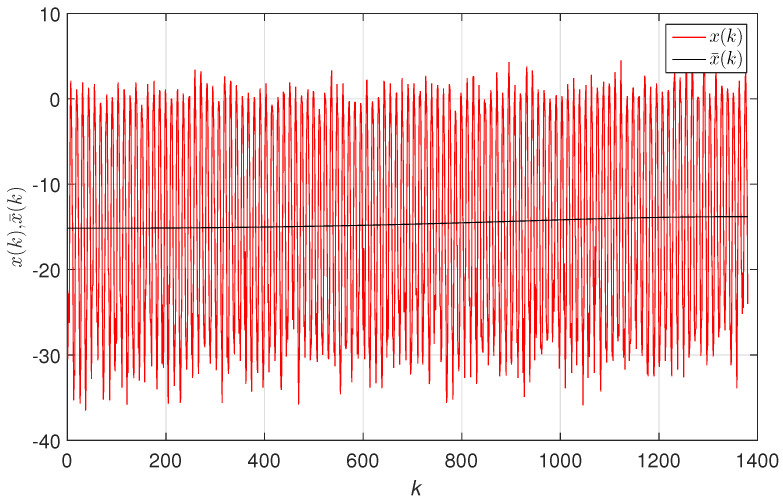
Monthly mean TTS, x(k), and trend approximation, $\bar{x}\left(k\right)$, for the TTS at the latitude/longitude coordinates $\left(71.25^{\circ },89.75^{\circ }\right)$.

Figure 2 depicts the original data, x(k), the IMF modes, $d_{l}\left(k\right)$, l=1,…,7, and the residuals, $m_{L}\left(k\right)$, obtained for a TTS of a station with latitude/longitude coordinates $\left(71.25^{\circ },89.75^{\circ }\right)$, following the procedure reported in [60]. Figure 3 illustrates the original data, x(k), and the trend, $\bar{x}\left(k\right)=m_{L}\left(k\right)$.

### 4.1. Lempel–Ziv Complexity of the TTS

**Figure 4:**
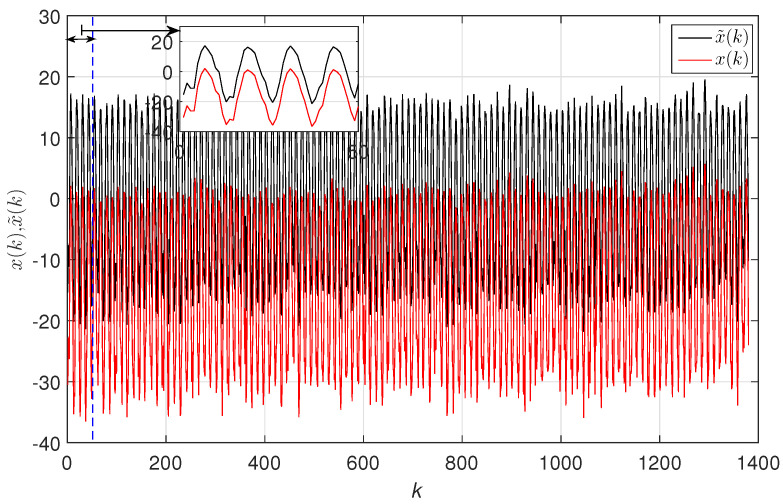
The detrending pre-processing of the TTS with latitude/longitude coordinates $\left(71.25^{\circ },89.75^{\circ }\right)$ by means of EMD.

Before determining the LZC, we first detrended the original TTS by calculating $\tilde{x}\left(k\right)=x\left(k\right)-\bar{x}\left(k\right)$, where $\bar{x}\left(k\right)$ was determined by EMD. Figure 4 exemplifies the detrending pre-processing ofn the TTS with latitude/longitude coordinates $\left(71.25^{\circ },89.75^{\circ }\right)$.

**Figure 5:**
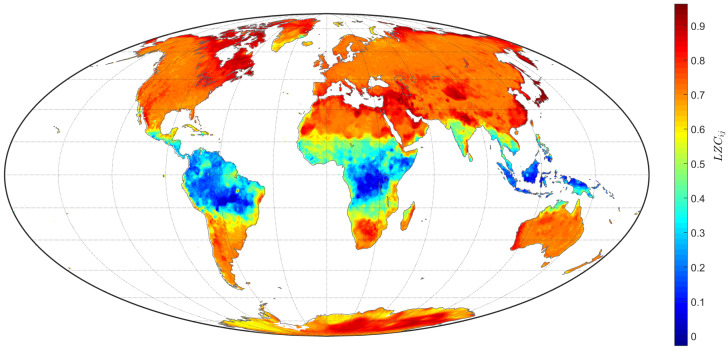
Contour map depicting $LZC_{ij}$ for the total L=85,690 TTS of the grid (i,j) on land.

The LZC algorithm shown in Section 2.1 was then applied to each $\tilde{x}\left(k\right)$ on the regular grid (i,j) covering land areas, totaling L=85,690 TTS. Figure 5 represents the $LZC_{ij}$ on a contour map. Using to the LZC index, we verified that the monthly mean TTS values in the Northern Hemisphere and in certain regions of South America and Australia are more complex than those in other regions of the Earth. This spatial variability of complexity suggests that climate dynamics are driven by distinct regional forcing processes.

### 4.2. Sample Entropy of the TTS

**Figure 6:**
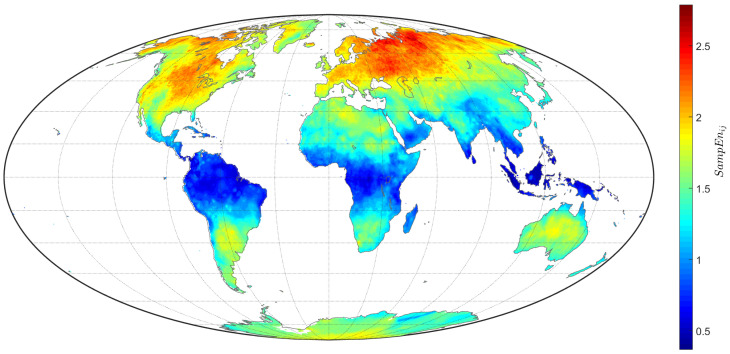
Contour map depicting $SampEn_{ij}$ for the total L=85,690 TTS of the grid (i,j) on land.

To calculate the SampEn, we applied the algorithm from Section 2.2 to the detrended TTS, $\tilde{x}\left(k\right)$, with m=2 and r=0.5, as discussed in Section 2.2. Figure 6 represents the $SampEn_{ij}$ for each TTS of the grid (i,j) on land.

To compare the results obtained with $LZC_{ij}$ and $SampEn_{ij}$ we calculated the two-dimensional correlation, *R*, as

(14) $$R=\frac{\sum_{i}\sum_{j}\left(LZC_{ij}-\langle LZC\rangle )\cdot (SampEn_{ij}-\langle SampEn\rangle \right)}{\sqrt{\sum_{i}\sum_{j}{\left(LZC_{ij}-\langle LZC\rangle \right)}^{2}}\cdot \sqrt{\sum_{i}\sum_{j}{\left(SampEn_{ij}-\langle LZC\rangle \right)}^{2}}},$$

where 〈·〉 denotes the mean of all values in a matrix. The correlation value obtained, R=0.97, quantified the similarities observed between Figure 5 and Figure 6, showing that both indices yield similar spacial patterns. The results thus suggest the existence of distinct regional climate forcing processes.

### 4.3. Harmonic Content of the TTS

In this sub-section, we propose an alternative index for measuring the complexity of TTS. The index, η, is given by the harmonics power ratio of the signals:

(15) $$\eta =\left(1-\frac{P_{h}}{P_{t}}\right)\times 100\%,$$

where

(16a) $$P_{h}=|a_{0}{|}^{2}+{\left|a_{1}\right|}^{2},$$

(16b) $$P_{t}=\frac{1}{T}\sum_{k=0}^{T-1}{\left|\tilde{x}\left(k\right)\right|}^{2},$$

when T=12 months.

Following Parseval’s theorem (10), $P_{h}$ and $P_{t}$ denote the signal power captured by the DC and first harmonic components, and the total power of $\tilde{x}\left(k\right)$, respectively. Therefore, η measures the signal power contained on the higher harmonics of $\tilde{x}\left(k\right)$.

**Figure 7:**
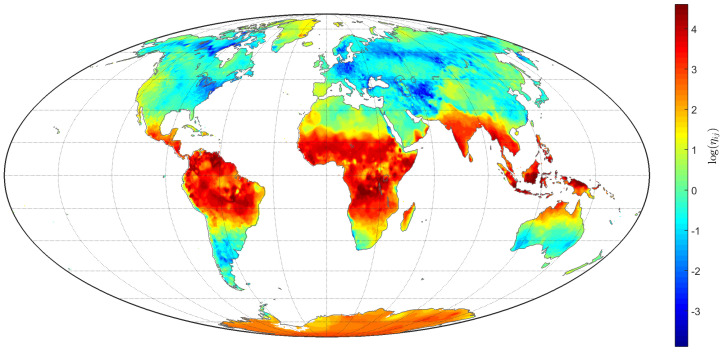
Contour map depicting $\eta_{ij}$ for the total L=85,690 TTS of the grid (i,j) on land.

Figure 7 represents the $\eta_{ij}$ for the TTS of the grid (i,j) on land. We verified that tropical and equatorial regions are characterized by larger values of η.

### 4.4. Fractal Dimension of the TTS

Here we calculate the FracDim for the phase portrait of the TTS. A *n*-dimensional dynamical system can be represented by a set of first-order differential equations governing *n* state variables. By knowing the state variables at time $k=k_{0}$ and the system inputs for $k\geq k_{0}$, the system behavior for $k\geq k_{0}$ can be determined. The state space consists of the set of all possible states, each one corresponding to a unique point. As *k* evolves, sequences of points describing trajectories in the state space are obtained. The set of trajectories is known as the state space portrait. The phase portrait (PP) corresponds to the two-dimensional state space representation [61,62].

**Figure 8:**
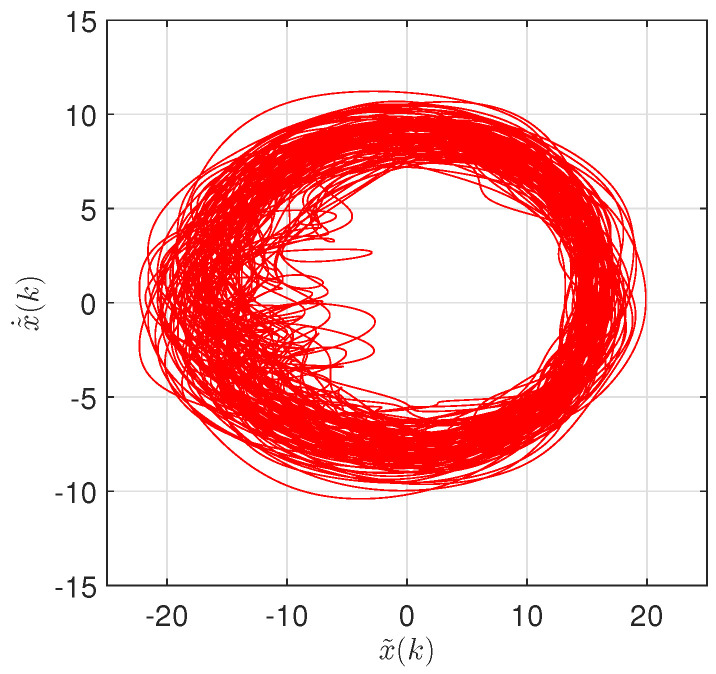
The phase portrait (PP) $\left(\tilde{x},\dot{\tilde{x}}\right)$ of the detrended TTS with latitude/longitude coordinates $\left(71.25^{\circ },89.75^{\circ }\right)$.

We consider the detrended TTS, $\tilde{x}\left(k\right)$, and its numerical time derivative, $\dot{\tilde{x}}\left(k\right)=\tilde{x}\left(k+1\right)-\tilde{x}\left(k\right)$, to be the state variables. Figure 8 represents the PP $\left(\tilde{x},\dot{\tilde{x}}\right)$ of the detrended TTS with latitude/longitude coordinates $\left(71.25^{\circ },89.75^{\circ }\right)$.

**Figure 9:**
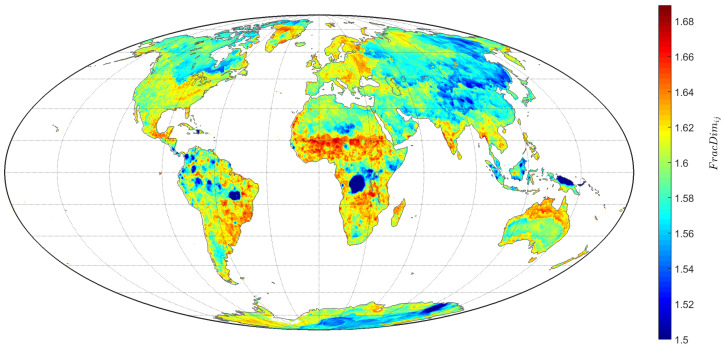
Contour map depicting $FracDim_{ij}$ for the total L=85,690 TTS of the grid (i,j) on land.

Figure 9 represents the $FacDim_{ij}$ for all TTS of the grid (i,j) on land.

## 5. Temporal Dynamics of Global Warming

**Figure 10:**
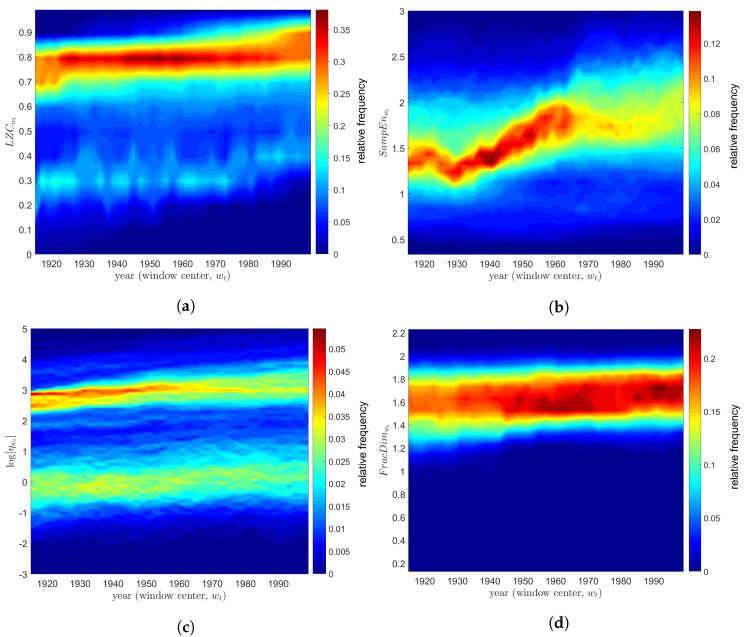
Contour maps of the histograms of the complexity indices versus $w_{t}$, t=1,…,W: (**a**) $LZC_{w_{t}}$; (**b**) $SampEn_{w_{t}}$; (**c**) $\log[\eta_{w_{t}}]$; (**d**) $FracDim_{w_{t}}$. Each year corresponds to the center of a time interval.

In this section we describe the study of the temporal complexity of global warming. In the first phase, TTS measured on land (within a total of L=85,690) was divided into W=43 time intervals, $w_{t}$, t=1,…,W, by means of a 30-year length sliding window, with a 28-year overlap. For each time interval, $w_{t}$, we calculated the *L*-dimensional vectors, $LZC_{w_{t}}$, $SampEn_{w_{t}}$, $\eta_{w_{t}}$ and $FracDim_{w_{t}}$, representing the complexity of the detrended TTS at the time intervals $w_{t}$, captured in the perspective of each of the four indices. Experiments demonstrated that the window adopted established a good compromise between time discrimination and statistical significance of the data within each time interval. In a second phase, for each indice and $w_{t}$ we determined the relative frequency histograms. Figure 10 depicts the histograms for the four indices versus $w_{t}$ on contour maps, where the years correspond to the center of the time windows. The $LZC_{w_{t}}$ and $\eta_{w_{t}}$ maps exhibited two modes that increased with $w_{t}$ almost monotonically. Regarding the $LZC_{w_{t}}$ index, higher modes were mainly due to the TTS in the regions of Antarctica, South Africa, East Australia, North Africa, the Middle East and some setentrional areas of America and Asia. On the other hand, lower modes were obtained from TTS located in the tropical regions of Earth. In the $\eta_{w_{t}}$, the higher and lower modes were due to TTS from equatorial and tropical regions, and to the TTS from North Australia, North Africa, East Asia and Northwest America. The $SampEn_{w_{t}}$ and $FracDim_{w_{t}}$ indices had single mode. For the $SampEn_{w_{t}}$, most TTS were from North Africa, South Asia, South America, Australia and Antarctica. For the $FracDim_{w_{t}}$, the main contribution was due to TTS from tropical regions.

In the follow-up, to unveil temporal patterns, we adopted a hierarchical clustering (HC) analysis and visualization trees. The main objective of HC is to group together objects that are similar to one another in some sense [63,64]. The input of HC is a W×W symmetric matrix $\left[d_{tu}\right]$, t,u=1,…,W, where $d_{tu}$ represents the Canberra distance between the time periods, $\left(w_{t},w_{u}\right)$ [29,65]:

(17) $$\begin{array}{cc} d_{tu}= & \sum_{l=1}^{L}\left(\frac{|LZC_{w_{t}}\left(l\right)-LZC_{w_{u}}\left(l\right)|}{|LZC_{w_{t}}\left(l\right)|+|LZC_{w_{u}}|\left(l\right)}+\frac{|SampEn_{w_{t}}\left(l\right)-SampEn_{w_{u}}\left(l\right)|}{|SampEn_{w_{t}}\left(l\right)|+|SampEn_{w_{u}}|\left(l\right)}+\right.\\ & \left.\frac{|\eta_{w_{t}}\left(l\right)-\eta_{w_{u}}\left(l\right)|}{|\eta_{w_{t}}\left(l\right)|+|\eta_{w_{u}}|\left(l\right)}+\frac{|FracDim_{w_{t}}\left(l\right)-FracDim_{w_{u}}\left(l\right)|}{|FracDim_{w_{t}}\left(l\right)|+|FracDim_{w_{u}}|\left(l\right)}\right). \end{array}$$

**Figure 11:**
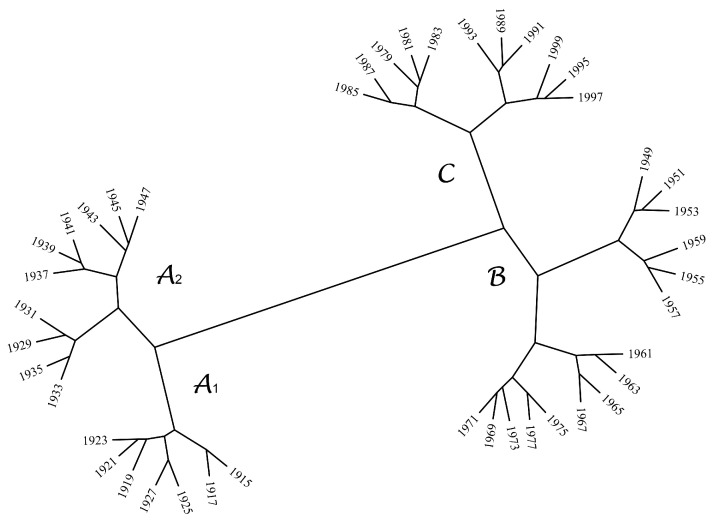
The hierarchical tree generated by the hierarchical clustering (HC) algorithm based on (17).

In the HC algorithm, the successive (agglomerative) clustering and average-linkage methods were adopted. Figure 11 depicts the corresponding graph generated by the software, PHYLIP (http://evolution.genetics.washington.edu/phylip.html). We identified three clusters of TTS complexity patterns, closely coincident to time periods characterized by different temperature trends [66], namely the warming period, A=[1915,1947], the cooling trend in the middle of the 20th century, B=[1947,1977], and the new warming period, C=[1977,1999]. Moreover, in A, the HC unveiled two groups, namely $A_{1}=\left[1915,1927\right]$ and $A_{2}=\left[1927,1947\right]$. In the clusters $A_{2}$, B and C, we noted the emergence of some smaller clusters. In $A_{2}$, we found subclusters composed of the years {1929,1931,1933,1935} and {1937,1939,1941,1945,1947}. In B, we verified the appearance of the groups {1949,1951,1953,1955,1957,1959} and {1961,1963,1965,1967,1969,1971,1973,1975,1977}. Finally, in C, there were {1979,1981,1983,1985,1987} and {1991,1993,1995,1997,1999}. These subclusters include between 4 and 6 time windows, suggesting some kind of periodicity. However, to the authors’ best knowledge, there have been no studies addressing these fluctuations with the standard perspective of the rise in temperature. Therefore, we question whether there is some hidden correlation between the increasing temperatures and their complexity that has somehow been overlooked in previous approaches. This open question will require additional studies with more detailed data analyses in time and space.

In summary, we verified that the complexity of the TTS is increasing, that a solid assessment is accomplished when a combination of several distinct measures is used, and that complexity is an important issue to consider when analyzing global warming.

## 6. Conclusions

The TTS embeds rich information contributed by a multitude of distinct factors at different scales. The complexity of the TTS is related to climate forcing factors. In this paper, information from worldwide land meteorological stations was processed and its complexity was calculated in space and time by means of different indices. The increasing complexity of the TTS in recent years demonstrates that there has been a change in the dominating factors that drive the climate. On one hand, we have natural forces, such as the orbital effect, solar radiation, volcanic eruptions, changes in land cover, greenhouse gas, and aerosols. On the other hand, we find anthropogenic factors, including the rise in greenhouse gas and aerosols caused by human activities, as well as sulfate air pollutants, reactive nitrogen, dust, urban heat islands, ozone change, and land-surfaces changes also due to human activities. Four complexity measures were adopted. However, no single measure captured all effects and a multi-measurement perspective led to a superior assessment of the system state. The resulting multidimensional set of measurements was synthesized by means of the HC visualization technique. The obtained clusters revealed clear patterns in the increasing complexity embedded in the TTS. More detailed quantification of these patterns by means of spatial statistics, namely spatial Fourier power spectrum, will be addressed in further research.
